# Nanoscale surface modifications to control capillary flow characteristics in PMMA microfluidic devices

**DOI:** 10.1186/1556-276X-6-411

**Published:** 2011-06-03

**Authors:** Subhadeep Mukhopadhyay, Susanta S Roy, Raechelle A D'Sa, Ashish Mathur, Richard J Holmes, James A McLaughlin

**Affiliations:** 1 Nanotechnology and Integrated Bio-Engineering Center, School of Engineering, University of Ulster, Jordanstown, Co Antrim, BT37 0QB, Northern Ireland, UK

## Abstract

Polymethylmethacrylate (PMMA) microfluidic devices have been fabricated using a hot embossing technique to incorporate micro-pillar features on the bottom wall of the device which when combined with either a plasma treatment or the coating of a diamond-like carbon (DLC) film presents a range of surface modification profiles. Experimental results presented in detail the surface modifications in the form of distinct changes in the static water contact angle across a range from 44.3 to 81.2 when compared to pristine PMMA surfaces. Additionally, capillary flow of water (dyed to aid visualization) through the microfluidic devices was recorded and analyzed to provide comparison data between filling time of a microfluidic chamber and surface modification characteristics, including the effects of surface energy and surface roughness on the microfluidic flow. We have experimentally demonstrated that fluid flow and thus filling time for the microfluidic device was significantly faster for the device with surface modifications that resulted in a lower static contact angle, and also that the incorporation of micro-pillars into a fluidic device increases the filling time when compared to comparative devices.

## Introduction

In recent years, microfluidics has become an indispensable component of microelectromechanical systems (MEMS) technology [1-3], with polymer devices establishing a greater role in the development of disposable microfluidic systems [4]. One such polymer is polymethylmethacrylate (PMMA) which is used in the fabrication of a wide variety of microfluidic devices [4,5], from micro-reactors [4] to high aspect ratio microstructures [6], blood filters [7], and waveguide devices [8]. Additionally, PMMA microfluidic systems may be fabricated using a wide range of techniques, including injection molding, hot embossing, laser photo-ablation, and X-ray lithography [3,4,6,7,9].

Passive capillary flow is an important consideration for disposable polymeric microfluidic devices [2,10-12], where flow can be modified by adjusting the surface wettability or by incorporating surface roughness features on the interior surface of the microchannels. In literature, mainly three surface engineering strategies have been developed that can directly change nanoscale surface properties of polymer. First, fabricating desired surface features by various micro fabrication techniques, such as lithographic, hot-embossing, etc. Secondly, vacuum-based thin film coating techniques can be used to modify surface properties. Finally, polymer surface can be modified by physical (such as plasma) and chemical routes. Recent reports have shown that the surface wettability for polymer devices can be varied by plasma treatment [13-16] and the coating of diamond-like carbon (DLC) film [17,18] on the microchannel surfaces with numerous simulations describing the effects of surface roughness on microfluidic flow [2,19-22]. The plasma treatment and DLC coating on polymer for the surface modification have been well studied by other researchers. Chemical modification technique is also useful on PMMA surface for microfluidic applications [23]. Also, pristine and UV-modified PMMA surfaces were used in microfluidic devices for cell transport applications [24]. However, to the best of our knowledge, the effect of these surface modifications on microfluidic flow is not well understood. As such this study on the effects of microfluidic flow is essential to aid the design and fabrication processes of polymer microfluidic systems.

In this study, we have fabricated PMMA microchannels incorporating a micropillar array structure and subjected the channel surfaces to both plasma modification and DLC coatings to study the effects of surface modification on surface energy and surface chemistries. The capillary flow (in terms of capillary meniscus position and filling time of the devices) was recorded as a series of video files, which were subsequently analyzed to correlate the flow behavior of the surface modification system.

## Experimental techniques

### Fabrication of PMMA microchannels

An SU8 stamp-on-silicon-wafer was fabricated using an SF100 maskless photolithograph system (Intelligent Micro Patterning, LLC, USA) using an established SU8 processing method [5,25,26]. Briefly, SU8 50 was coated onto the silicon substrate using a spin coater at 1000 RPM to make the SU8 stamp, patterned following a soft bake by exposure to UV light at an intensity of 310 μW/cm^2 ^for 25 s, and developed following post-exposure bake in EC solvent for 10 min, following which the stamp was hard baked. The PMMA channels were fabricated from the stamp using a hot embossing system (EVG520, EVG Group, Austria) [5-7,9] operating at 125°C and 10 kN for 2 min. Finally, a direct bonding technique was used [5,27] to seal the PMMA devices to a PMMA lid. The bonding temperature and pressure used were 90°C and 10 kN, respectively, for bonding time of 4 min.

**Figure 1:**
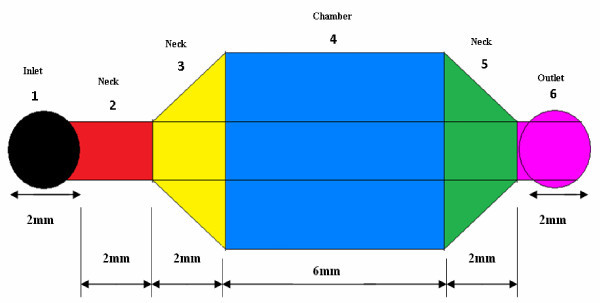
**Schematic (top view) of the microfluidic device, with proper length of each region**.

Figure 1 represents the schematic of the microchannels used to study the effects of different surface properties (surface wettability and surface roughness) on flow through microfluidic channels, with amaranth dye (Sigma Aldrich, UK) used to aid visualization of the water meniscus. The microchannel dimensions following hot embossing were verified using a Dektak 8 profilometer (Vecco Instruments, Santa Barbara, CA, USA). The microchannel design [2,11] shown in Figure 1 can be described as follows Regions 1 and 6 are circular inlets and outlets with a diameter of 2 mm. Region 2 has uniform width of 1.5 mm, while the width of region 3 increases from 1.5 to 5.0 mm. Region 4 (the chamber) has the length of 6 mm and width of 5 mm, and region 5 decreases from 5 to 1.5 mm in width. Each of the regions 2, 3, and 5 has a length of 2 mm along the channel axis and the height of the microchannel across all regions is 33 μm.

**Figure 2:**
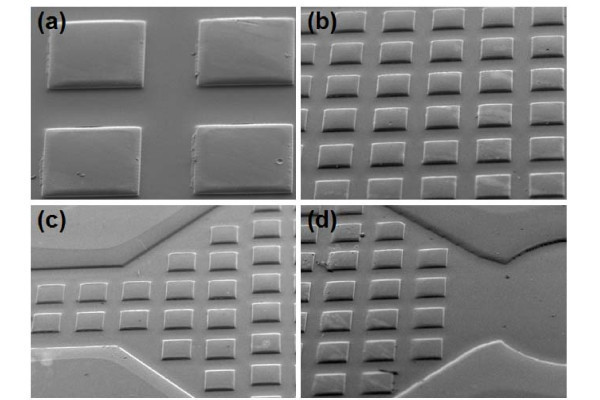
**SEM images of a microfluidic channel with 300 μm pillars**. (a) Closure image of 300 μm pillars; (b) arrays of 300 μm pillars; (c) 300 μm pillars in the regions just after the inlet; and (d) 300 μm pillars in the region just before the outlet.

The PMMA microchannels described above were fabricated in two categories: (i) planar faces with no micro-pillar structures present on any of the walls of the device, (ii) planar faces on all but the lower wall, where micro-pillar features were hot-embossed. The micro-pillars were used as surface roughness elements [2,22] with a height of 15 μm, and were fabricated from the beginning of region 3 and to the end of region 5, as arrays of 100 m (1.6% increase per mm^2^), 200 m (1.9% increase per mm^2^), or 300 m pillars (1.8% increase per mm^2^), with an inter-pillar separation (horizontal distance between any two subsequent micropillars) of 200 μm. Figure 2 shows example of SEM images of microchannel containing pillars of 300 μm in width.

### Surface modifications on the PMMA microchannel surfaces

We utilized four different methods to modify the pristine PMMA surfaces following heat and pressure treatment by hot embossing, but prior to thermal bonding, to modify the surface properties and wettability of the devices. The methods can be described briefly as follows:

#### Air dielectric barrier discharge (DBD) processing

Pristine PMMA surfaces were modified using an air DBD [13-16] treatment, adjusting the lift length to 740 mm with a 20% ramp over five cycles. After the plasma treatment on the PMMA surface, the sample was stored in air for 48 h and the static water contact angle was measured as 58.1. After 5 days of that plasma treatment, the contact angle on the same plasma-treated surface was subsequently measured as 44.3.

#### Nitrogen plasma treatment

Pristine PMMA surfaces were treated using an N_2 _plasma in a Plasma Enhanced Chemical Vapor Deposition (PECVD) system [18]. The vacuum pressure in PECVD chamber was maintained as 5.2 × 10^-6 ^Torr, and the sample was cleaned with Ar at 60 sccm with a working pressure of 1.5 × 10^-2 ^Torr. The treatment time was 5 min for the gas mixture of Ar (5 sccm) and N_2 _(10 sccm) and the working pressure during deposition was maintained at 3.0 × 10^-3 ^Torr.

#### Coating of hydrogenated amorphous carbon (a-C:H)

Pristine PMMA surfaces were coated with hydrogenated amorphous carbon (a-C:H) using a PECVD system [17]. The vacuum pressure in PECVD chamber was maintained as 4.6 × 10^-6 ^Torr, and the sample was cleaned with Ar at 60 sccm with a working pressure of 1.6 × 10^-2 ^Torr. The deposition time was 5 min for the gas mixture of Ar (10 sccm) and C_2_H_2 _(20 sccm) with a working pressure of 7.7 × 10^-3 ^Torr.

#### Coating of Si-doped hydrogenated amorphous carbon (a-C:H)

Pristine PMMA surfaces were coated with Si-doped a-C:H using a PECVD system [17]. The vacuum pressure in PECVD chamber was maintained as 6.2 × 10^-6 ^Torr, and the sample was cleaned with Ar at 60 sccm at a working pressure of 1.8 × 10^-2 ^Torr. The deposition time was 5 min for the gas mixture of Ar (5 sccm), C_2_H_2 _(10 sccm) and TMS (5 sccm) in 8.9 × 10^-3 ^Torr chamber pressure.

### Characterization techniques

#### Surface characterizations

The surface of PMMA was not rinsed with IPA and DI water prior to contact angle analysis. However, rinsing PMMA surfaces by IPA and DI water may alter contact angles [28]. Two different liquids were used to carry out the contact angle measurements: ultra-pure water (MilliQ^®^) and Ethylene Glycol (Sigma-Aldrich, Gillingham, UK). Static contact angle were measured (using CAM 2000, KSV Instrument Ltd., Helsinki, Finland) by the sessile-drop method at room temperature (approximately 25°C). A 5 μl droplet of the liquid was deposited on the surface of the sample, and immediately after stabilization, an image of the droplet was captured. The profile of the droplet was automatically fitted with the CAM 2000 software using a Young Laplace approach. At least ten readings were performed per sample type and the corresponding average values and standard deviations were recorded. We have studied the surface chemistry and roughness of the unmodified and modified PMMA surfaces by X-ray photo electron spectroscopy (XPS) and atomic force microscopy (AFM), respectively.

#### Fluidic measurements for the microfluidic devices

Fluid flow in the sealed devices was recorded using a CMOS camera capturing video at 25 frames per second (fps) which corresponded to a frame resolution of 0.04 s/frame. The air-water interface velocity was measured from the recorded video clips [29] and the air-water interface velocity and the filling time (time for the meniscus to travel from the inlet to outlet of any particular device) were calculated by measuring the interface position change over a corresponding time interval [2,5]. The laboratory temperature and humidity at the time of recording were in the order of 25°C and 28%, respectively. The flow was visualized using amaranth dyed water, with 15 μl amount of working liquid volume dispensed to minimize the entrance effect in each system. The meniscus movements were measured along the center line of the chamber.

## Results and discussion

### Surface energy, surface chemistry, and surface roughness

The static contact angles of dyed water and ethylene glycol on pristine PMMA and modified flat surfaces is shown in Table 1 with the static contact angles of ethylene glycol used to calculate the surface energies. As such, two groupings of static contact angles of water have been observed (Table 1):

**Table 1:** Static contact angles of dyed water and ethylene glycol on different surfaces

Surface types	Static contact angles (°)	
	Dyed water	Ethylene glycol
Air DBD processed PMMA	44.35	25.25
Nitrogen plasma-treated PMMA	52.13	47.70
Undoped DLC-coated PMMA	72.66	20.26
Control PMMA	79.97	48.77
Si-doped DLC-coated PMMA	81.25	39.27

(i) 72.6 for hydrogenated amorphous carbon (a-C:H) coated surface, 79.9 for pristine PMMA surface and 81.2 for Si-doped a-C: H coated surface;

(ii) 44.3 for air DBD processed PMMA surface and 52.1 for Nitrogen plasma-treated PMMA surface.

The plasma treatment processes were shown to significantly reduce static contact angle from 79.9 to between 52.1 and 44.3, data that supports results presented in previous reports on DLC [30] and plasma-treated PMMA [31] surfaces. Additionally, in the literature, surface wettability (hydrophobicity or hydrophilicity) has been defined using the static water contact angle [29,32-35] method, and as such the plasma treatment process has been shown to considerably increased surface wettability-a feature which could be used to modify microfluidic flow in PMMA microchannels. The thickness of both undoped and Si-doped DLC coatings was measured as 70 nm by surface stylus profilometer.

**Figure 3:**
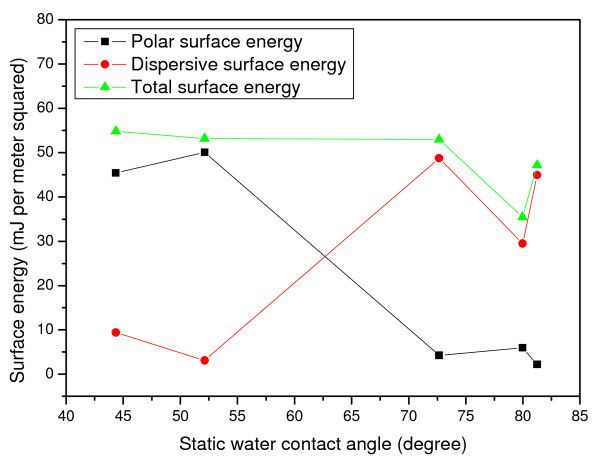
**Surface energy versus static water contact angle**.

Static contact angles for water and ethylene glycol were used to evaluate the dispersive and polar components of surface energy using the following relationship [36]:(1)

where *θ *is the contact angle of the liquid on the solid surface; *γ*_s_^d ^and *γ*_s_^p ^are the dispersion and polar components of surface free energy of the solid surface, respectively; *γ*_l_is the surface free energy of the liquid; *γ*_l_^d ^and *γ*_l_^p ^are the dispersion and polar components of surface free energy of the liquid surface, respectively. The calculated surface energies as a function of static water contact angle are shown in Figure 3, where in general, the static water contact angle decreases as the polar component of surface free energy (solid surface) increases [37-40]. Figure 3 also illustrates that the surface energy for both PMMA and DLC-coated PMMA were similar; however, Si-doped DLC demonstrated an increased surface energy when compared to pristine PMMA.

**Figure 4:**
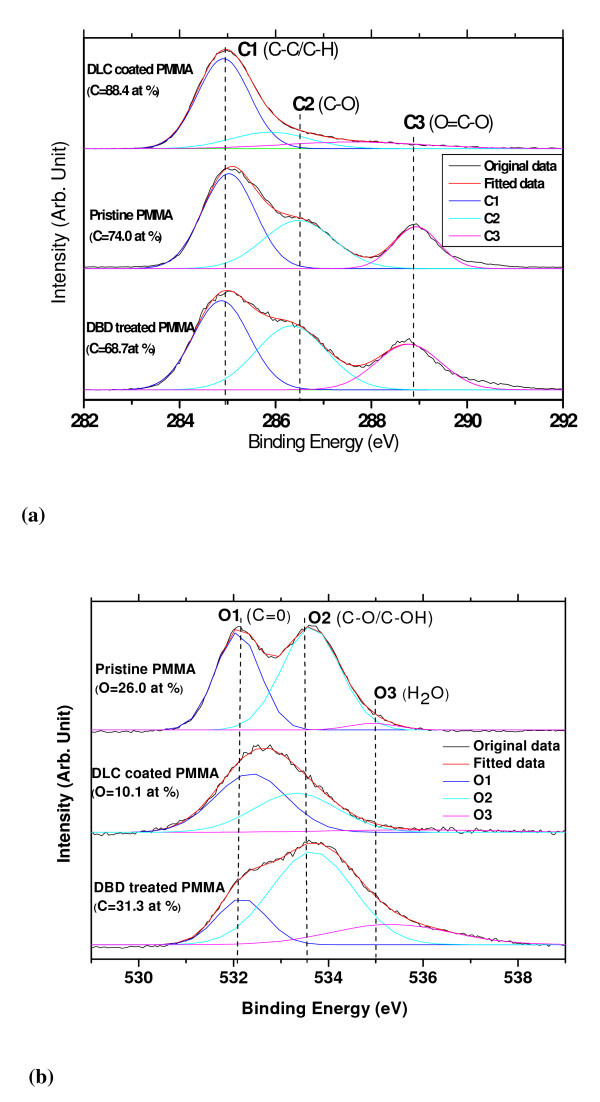
**Curve fitted XPS spectra for three surfaces**. (a) C 1s (b) O1s.

**Figure 5:**
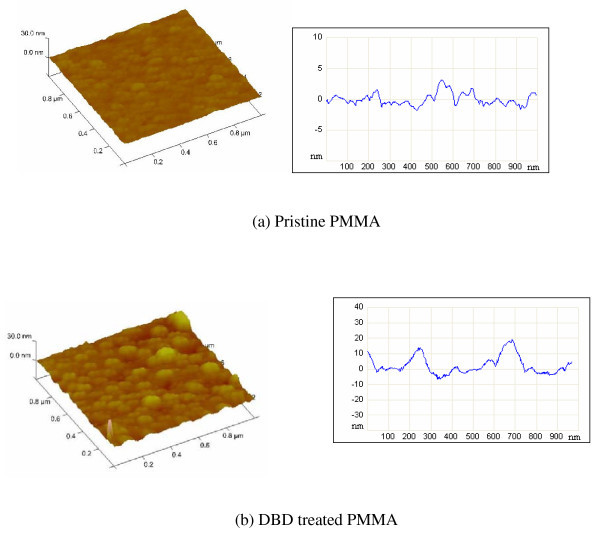
**AFM images and line profiles for (a) pristine PMMA surface; (b) DBD treated PMMA surface**.

The atomic percentage of chemical species and change to the chemical bonding configurations were probed by X-ray photoelectron spectroscopy (XPS). The corresponding C1s and O1s spectra for pristine, DBD modified, and a-C:H-coated PMMA surfaces are given in Figure 4a,b, respectively. For all the samples studied, the C1s envelope is curve fitted into three components [41-43] at binding energies of 285.0 eV (C-C/C-H), 286.7 eV (C-O), and 289.0 eV (O = C-O). The O1s envelope can be fitted with three peaks at 532.2 eV (C = O), 533.7 eV (C-O/C-H), and 535.0 eV (H_2_O), respectively [41,42]. The DBD treatment of PMMA clearly shows a loss of alkyl components (C-C/C-H) with a prominent increase in the oxidative groups and generates more hydroxyl components (Figure 4). In general, the DBD atmospheric pressure plasma treatment method generates radicals which, in the absence of other reactants, combine with oxygen from the environment to create oxidative functionalities such as peroxides and hydroperoxides on the polymer surface. The reactive oxygen groups slowly decompose to form more stable oxidative groups such as hydroxyls. A detailed explanation of the oxidation of PMMA observed by XPS analysis after DBD modification has been previously reported by us [44]. In the case of DLC-coated samples, they had less oxygen and hydrocarbon groups. The changes in surface topography due to the surface modifications were probed using an atomic force microscope (AFM). The AFM results suggested that the air DBD process increased surface roughness of pristine PMMA. Both the average and the RMS roughness values increased from 0.71 and 0.90 nm to 2.40 and 3.40 nm, respectively, as shown in Figure 5. It is reported that for surfaces with contact angle less than 90°, the increase of surface roughness reduces the static water contact angle [45]. We have observed that the static water contact angle was much less on the air DBD-treated PMMA than that on the pristine PMMA surface. This type of surface engineering is quite useful in tuning the wettability of the polymer surfaces.

### Theoretical background of capillary flow in rectangular microchannel

In a recent report, Waghmare and Mitra[46,47] derived the pressure field distribution in the capillary meniscus at the entrance of a rectangular microchannel considering semi-cylindrical control volume. In another recent report, Waghmare and Mitra[46,47] investigated the numerical solutions of flow front penetration in vertically oriented rectangular microfluidic channels considering finite reservoir at the inlet to study the effect of gravity along with capillarity. In our experimental study, the control volume was not semi-cylindrical. Also, the capillary flow was not significantly gravity assisted as the microchannel was not vertically oriented. Therefore, we used a simplified model to compare the experimental results of capillary meniscus movements with analytical solutions [48]. Interface between solid-liquid-gas has special effect on the speed of the fluidic flow in micro dimension. The instantaneous position (penetration depth) of the capillary meniscus can be derived from incompressible Navier-Stokes equation using the continuity equation and momentum equation [48]:(2)

where *D *is the diffusion coefficient and *L*_o _= *L*(0).(3)

where *ρ*, *G*, *H*, *h*, and *μ *are density, gravitational acceleration, height of the liquid reservoir at the inlet, microchannel height, and viscosity of liquid, respectively. The surface tension term (*γ*_sa _- *γ*_sl_) can be calculated from Equation 3. *γ*_sa _is the surface tension of the solid-air interface and *γ*_sl _is the surface tension of the solid-liquid interface. In this study, 1st term in right-hand side of Equation 3 has been neglected as *H *~ 0 for surface driven flow.

Moreover, capillary pressure (Δ*P*_cap_) at the solid-liquid-gas interface can be given by the following equation [12,49]:(4)

where, *γ*_lg _is the water-air surface tension per unit area. *θ*_2 _is the water-top wall contact angle and *θ*_1 _is the contact angle of water on any other wall of the microchannel. The water meniscus will propagate in microchannel by capillary action if Δ*P *has positive value [12], and in our experiments, we have observed that the air-water meniscus movement was slower in the microchannel of higher contact angle, indicating that θ_1 _(surface wettability reduced) was increased while maintaining *w*, *h*, and *θ*_2_. For higher contact angles fluid flow is significantly slower than for low contact angles, a conclusion supported by Equation 4.

### Effect of surface wettability on microfluidic flow of dyed water

**Figure 6:**
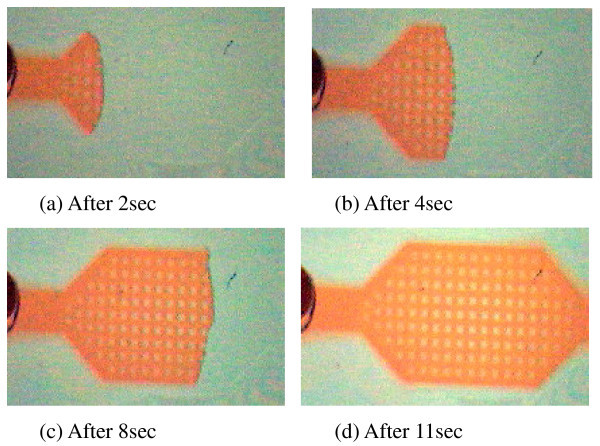
**Snap shot images of capillary filling of air DBD processed microchannel containing 300 μm pillar**. **(a) **After 2 s, **(b) **After 4 s, **(c) **After 8 s, **(d) **After 11 s.

**Figure 7:**
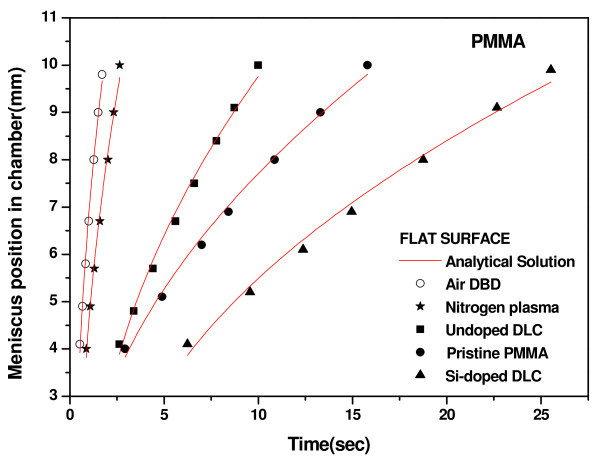
**Experimental and analytical meniscus position-time plots for pristine PMMA and modified flat surfaces**.

**Figure 8:**
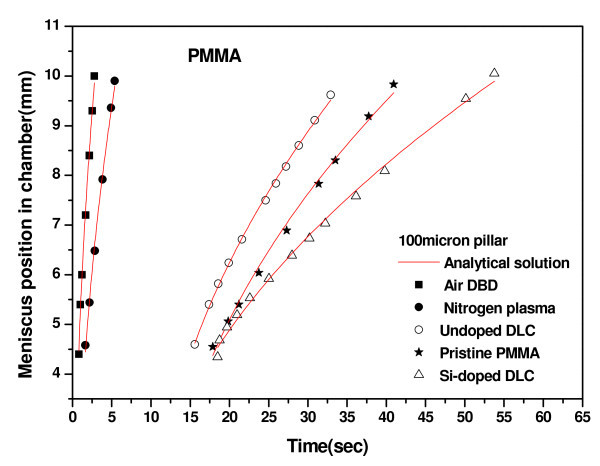
**Experimental and analytical meniscus position-time plots for pristine PMMA and modified pillars surfaces**.

**Figure 9:**
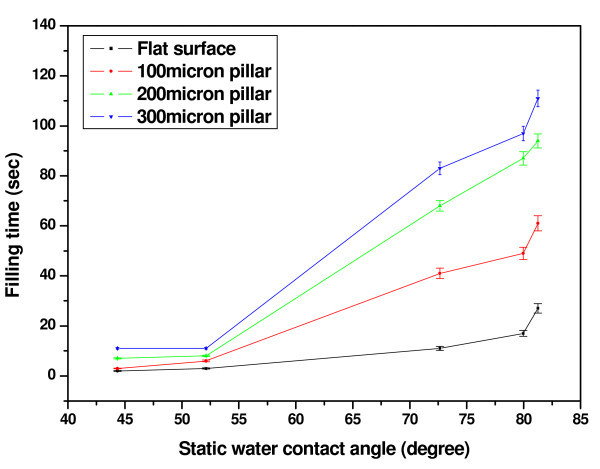
**Variation of filling time with static water contact angle**.

The air-water interface position and the filling time (time required for the meniscus to travel from the inlet to outlet of the device) were determined from the video files captured using the CMOS camera. A representative sequence of frames following the meniscus movement of dyed water on the air DBD processed microchannel surface containing 300 μm pillar is shown in Figure 6. While, Figure 7 presents the meniscus position-time graphs for the device containing no pillars on the channel wall and Figure 8 presents the data for the device containing 100 μm pillars on the bottom of the microchannel. The variation in filling time as a function of contact angle is shown in Figure 9, and the following observations can be made from the data presented in Figure 7 to Figure 9:

(i) The filling time is higher for the microchannel surfaces with higher static water contact angle for any category of devices;

(ii) In Figures 7 and 8, the meniscus movement was faster for plasma-treated surfaces at all positions in the microchannels than on the pristine and DLC-coated PMMA surfaces. A similar trend of meniscus movement was shown the variation in pillar dimensions;

(iii) The linear relationship was shown between pillar dimension (none, 100, 200, 300 m) and flow velocity in the microchamber;

(iv) The effect of pillar side length was more pronounced on the surfaces of higher static water contact angle.

We also compared our experimental results with analytical simulation using Equation 2. Excellent fitting were obtained for all data. We have determined the values of diffusion coefficient for each microfluidic flow on different categories of surfaces (Figure 1). The diffusion coefficient was higher for the microfluidic flow on the surface of lower static contact angle (Table 2). We have evaluated the surface tension term (*γ*_sa _- *γ*_sl_) from Equation 3 from the results of analytical simulation. Surface tension term (*γ*_sa _- *γ*_sl_) was directly proportional to diffusion coefficient (*D*). The surface tension term was higher for the plasma-treated surfaces than other surfaces (Table 2). Yang et al. [48] has also shown that the surface tension term was higher for the surface of lower contact angle.

The variation of static water contact angle in the microchannel structure has been shown to have a significant influence on the speed of microfluidic flow, indeed Sultana et al. [50] demonstrated the dependence of microfluidic flow on surface wettability, and observed that the flow rate decreased with the decrease of surface wettability [50] between static contact angles of 20 and 89°. As lower static water contact angle is a measurement of higher surface wettability [30,33,35,36], so the meniscus movement was faster on the microchannel surface of with lower static contact angle [30]. Especially, when Suk and Cho [12] observed, the meniscus movement became significantly slower when the hydrophobic patterns were created in polymeric microchannel.

In this paragraph, we attempted to correlate fluid flow characteristic with surface properties and device structures. The lower filling time on plasma-treated surfaces was due to the higher polar surface energy due to plasma treatment (Figure 3). The surface chemistry studies revealed higher oxygen content and hydroxyl species on DBD processed PMMA surfaces (Figure 4b) and that may be one of the reasons of higher speed of fluid flow than on pristine PMMA surface. The higher surface roughness on DBD processed PMMA (Figure 5) may be another reason of higher speed of water flow than that on pristine PMMA surface [45]. The geometry of the microchannel can also play a significant effect on microfluidic flow. We have modified the speed of microfluidic flow by varying the surface wettability and designed surface roughness on the bottom wall of microchannel, and Saha et al. [11] theoretically predicted that the channel walls and pillars would have significant effects on fluid flow, with the contributions being 1-3% for the side walls, 5-13% for the pillars and 85-89% from the top and bottom walls. In our study, we modified the bottom and side wall surfaces to change the surface wettability (measured as static contact angle on the surface), however the surface wettability on the top wall was constant for each device. Since the surface area to volume ratio is very high in microchannels, the surface wettability has a significant effect on microfluidic flow, and as a result a small change of contact angle leads to a larger change in capillary forces, thus making a significant change in filling time (Figure 9).

### Effect of micropillar side length on the microfluidic flow of dyed water

In a pressure-driven flow system, Wang et al. [22] demonstrated that the Poiseuille number increased with increasing size of the roughness elements, while the mass flow rate can be seen to decrease with the increasing Poiseuille number [51]. So, the mass flow rate was lower with increasing size of surface roughness elements. In our experimental study on capillary flow, PMMA micropillars were used to enhance surface roughness of the system. Pillar height and spacing were constant but the side length was varied to study the effect of micropillar dimension on microfluidic flow. Figure 9 illustrates that the filling time of the capillary meniscus was higher in the microfluidic device integrated with micropillars of higher side length. So, the speed of the capillary meniscus was lower in the device integrated with higher side length micropillars.

Rawool et al. [52] observed that the microfluidic flow was slower in microchannel containing obstructions of larger height on the channel wall due to enhancement of the friction factor, which corroborates our observations that the microfluidic flow of dyed water was slower for the larger side lengths of PMMA pillars, with the change in surface area resulting from the micropillars being 1.6-1.9%. As the surface area to volume ratio increases the average meniscus velocity can be seen to decreases for surface-driven flow in microchannels [11]. Saha et al. [11] used computer simulations to predict that the effect of pillars on speed and filling time would be significantly higher for higher of contact angles. This is supported by our work, where we have observed a similar behavior (Figure 9). In other words, the pillars have highly significant effects on dyed water flow on the surfaces of higher static contact angles but less significant effects for lower static contact angles. The diffusion coefficient was higher for the microfluidic flow through the microchannel containing micropillars of smaller side length for each of the pristine and modified surfaces (Table 2). Also, the surface tension term (*γ*_sa _- *γ*_sl_) was higher for the microchannel surface containing micropillars of smaller side length for each of the pristine and modified surfaces (Table 2).

**Table 2:** Values of diffusion coefficients and surface tension parameters from analytical simulation on each position-time curve of all the microfluidic flow

Surface modification	Surface structure	Diffusion coefficient, *D *(ρm^2 ^s^-1^)	*γ*_sa _- *γ*_sl_ N/m
Air DBD processed	Flat surface	66153420	0.004330
	100 μm pillar	38677500	0.002531
	200 μm pillar	23777420	0.001556
	300 μm pillar	12537020	0.000820
Nitrogen plasma treated	Flat surface	45091690	0.002951
	100 μm pillar	20411460	0.001336
	200 μm pillar	12500060	0.000818
	300 μm pillar	11718310	0.000767
Control PMMA	Flat surface	6337320	0.000414
	100 μm pillar	3219800	0.000210
	200 μm pillar	2142380	0.000140
	300 μm pillar	1477070	0.000096
Undoped DLC coated	Flat surface	10878150	0.000712
	100 μm pillar	3993380	0.000261
	200 μm pillar	2983690	0.000195
	300 μm pillar	1782760	0.000116
Si-doped DLC coated	Flat surface	4044020	0.002646
	100 μm pillar	2058300	0.000134
	200 μm pillar	1449660	0.000094
	300 μm pillar	1175890	0.000076

## Conclusions

In this study, we have shown that the microfluidic flow behavior can be significantly varied by two simple ways; first, by the plasma treatment on PMMA microchannel; secondly, by the variation of designed surface roughness (micropillars). Comprehensive analysis of surface energy and surface chemistry studies revealed the reasons for the change of fluid flow behavior in microchannel. The static water contact angle on PMMA surfaces being reduced significantly by a plasma treatment processes. The polar surface energy was shown to be higher for the surfaces of lower static water contact angle and the oxygen content, hydro-carbon groups, and surface roughness were notably higher on DBD processed PMMA than for pristine PMMA. The pristine and modified surfaces can be classified into two different groupings of wettabilities, determined by static water contact angles, above 70 and near to 50°. The dyed water flow was faster on the surface of lower static contact angle due to higher wettability, and the effect of pillar side length shown to be more significant on the surface of higher static water contact angle. This type of surface engineering of any polymeric material can be widely used in variety of applications such as microfluidic and bio-engineering.

## Abbreviations

AFM: atomic force microscope; DLC: diamond-like carbon; fps: frames per second; MEMS: microelectromechanical systems; PECVD: plasma enhanced chemical vapor deposition; PMMA: polymethylmethacrylate; XPS: X-ray photo electron spectroscopy.

## Competing interests

The authors declare that they have no competing interests.

## Authors' contributions

SM fabricated the microfluidic devices and analysed the fluid flow phenomena. SM, SSR and RAD performed the surface modifications. RAD, AM and RJH carried out the SEM, AFM and XPS studies. JM designed the study and participated in its coordination. SM and SSR wrote the manuscript. All authors read and approved the final manuscript.
